# Policy relevant health related liveability indicator datasets for addresses in Australia’s 21 largest cities

**DOI:** 10.1038/s41597-023-02013-5

**Published:** 2023-02-25

**Authors:** Carl Higgs, Melanie Lowe, Paula Hooper, Suzanne Mavoa, Jonathan Arundel, Lucy Gunn, Koen Simons, Billie Giles-Corti

**Affiliations:** 1 RMIT University, Centre for Urban Research, Melbourne, 3000 Australia; 2 Australian Urban Design Research Centre, University of Western Australia, Perth, 6009 Australia; 3 Melbourne School of Population & Global Health, University of Melbourne, Parkville, 3010 Australia; 4 Environmental Public Health Unit, Environment Protection Authority Victoria, Melbourne, VIC 3001 Australia

**Keywords:** Risk factors, Sustainability

## Abstract

Measuring and monitoring the spatial distribution of liveability is crucial to ensure that implemented urban and transport planning decisions support health and wellbeing. Spatial liveability indicators can be used to ensure these decisions are effective, equitable and tracked across time. The 2018 Australian National Liveability Study datasets comprise a suite of policy-relevant health-related spatial indicators of local neighbourhood liveability and amenity access estimated for residential address points and administrative areas across Australia’s 21 most populous cities. The indicators and measures encompass access to community and health services, social infrastructure, employment, food, housing, public open space, transportation, walkability and overall liveability. This national ’baseline’ liveability indicators dataset for residential address points and areas can be further linked with surveys containing geocoded participant locations, as well as Census data for areas from the Australian Statistical Geography Standard. The datasets will be of interest to planners, policy makers and researchers interested in modelling and mapping the spatial distribution of urban environmental exposures and their relationship with health and other outcomes.

## Background & Summary

Identifying, measuring and monitoring spatial indicators of urban liveability is key for planning of healthy, sustainable cities by all levels of government across diverse global contexts^1–4^. In particular, benchmarking and monitoring urban liveability is crucial to ensure planning decisions are both effective and equitable^5,6^. The 2018 Australian National Liveability Study (referred to hereafter as ‘the study’) was undertaken to calculate a suite of policy-relevant health-related spatial indicators of local neighbourhood liveability and amenity access for residential address points across Australia’s 21 largest cities, in terms of their usual resident population: the state and territory capital cities of Adelaide, Brisbane, Canberra, Darwin, Hobart, Melbourne, Perth and Sydney; and the regional cities of Ballarat, Bendigo, Cairns, Geelong, Launceston, Mackay, Sunshine Coast, Toowoomba, Townsville, Wollongong; and regional conurbations of Albury – Wodonga, Gold Coast – Tweed Heads and Newcastle – Maitland. Collectively the urban portions of these 21 cities account for approximately 75% of Australia’s population^7^. The resulting 14 datasets (Table 1) comprise a rich census of policy-relevant, health-related built environment measures and have already supported a broad range of outputs that enable policymakers and researchers to monitor local neighbourhood liveability, and research studies evaluating the extent to which built environment features are associated with the health and wellbeing of diverse cohorts including children, adults, older adults and people living with disability^8^.

**Table 1:** Australian National Liveability Study 2018 datasets.

File and description	Records	Format	Size	Data dictionary
*hlc_ntnl_liveability_2018_address_points_indicators_epsg7845.csv* Liveability indicators for residential locations (address points in urban Mesh Blocks with dwellings at 2016 Census)	6,536,400	CSV	5.1 GB	1 - Address point indicators.csv
*hlc_ntnl_liveability_2018_address_points_distance_closest_epsg7845.csv* Estimates for distance in metres along pedestrian network to the closest of a range of destination types for residential locations (address points in urban Mesh Blocks with dwellings at 2016 Census)	6,536,400	CSV	3.9 GB	2 - Address distance to closest.csv
*hlc_ntnl_liveability_2018_address_points_distance_arrays.csv* Arrays of estimates for distance in metres along pedestrian network to all destinations (within 3200 m and the closest) across a range of destination types, for residential locations (address points in urban Mesh Blocks with dwellings at 2016 Census)	6,536,400	PSV	11.2 GB	3 - Address destination array.csv
*hlc_ntnl_liveability_2018_Mesh_Block_2016.csv* Mesh Block averages of residential liveability indicators and distance to closest estimates, with dwelling and person counts as well as area linkage codes to support aggregation to larger area scales (optionally with weighting; recommended)	183,075	CSV	427 MB	4 - Area aggregate indicators.csv
*hlc_ntnl_liveability_2018_sa1_2016.csv* Liveability indicators for dwellings, aggregated for Statistical Areas Level 1 (SA1)	39,966	CSV	104 MB	4 - Area aggregate indicators.csv
*hlc_ntnl_liveability_2018_sa2_2016.csv* Liveability indicators for dwellings, aggregated for Statistical Areas Level 2 (SA2)	1,498	CSV	4.2 MB	4 - Area aggregate indicators.csv
*hlc_ntnl_liveability_2018_sa3_2016.csv* Liveability indicators for dwellings, aggregated for Statistical Areas Level 3 (SA3)	223	CSV	661 kB	4 - Area aggregate indicators.csv
*hlc_ntnl_liveability_2018_sa4_2016.csv* Liveability indicators for dwellings, aggregated for Statistical Areas Level 4 (SA4)	63	CSV	187 kB	4 - Area aggregate indicators.csv
*hlc_ntnl_liveability_2018_ssc_2016.csv* Liveability indicators for dwellings, aggregated for Suburbs	3,101	CSV	8.49 MB	4 - Area aggregate indicators.csv
*hlc_ntnl_liveability_2018_lga_2016.csv* Liveability indicators for dwellings, aggregated for Local Government Areas	170	CSV	502 kB	4 - Area aggregate indicators.csv
*hlc_ntnl_liveability_2018_region.csv* Liveability indicators for dwellings, aggregated for cities	21	CSV	63.4 kB	4 - Area aggregate indicators.csv
*hlc_ntnl_liveability_2018_gtfs_20191008_20191205_daytime_tidy_transit_headway_analysis.csv* GTFS transport stops headway analysis of day time weekday public transport service frequency between 8 October 2019 to 5 December 2019, with WKT geometry	111,593	CSV	10.2 MB	5 - Public transport frequency.csv
*hlc_ntnl_liveability_2018_aos_public_osm.csv* Areas of open space with at least partial public access, as identified using OpenStreetMap, with WKT geometry for public geometry, water geometry and overall geometry as well as JSON attributes (including public area) and list of co-located amenities within 100 m (including public toilets)	69,891	CSV	230 MB	6 - Public open space.csv
*hlc_ntnl_liveability_2018_od_aos_jsonb.tsv* JSON list of identifiers and distances of areas of open space for residential address points identified as having areas of open space accessible within 3200 m. This dataset is indexed by the residential address point identifier, supporting linkage with attributes from the main address indicator dataset.	6,535,982	TSV	17.5 GB	7 - AOS within 3200 m.csv

The selection of the health-related and policy-relevant indicators was informed by a comprehensive program of research that examined associations between the built environment and several health and wellbeing outcomes^9–18^. The selected indicators encompassed facets of urban liveability that influence one’s capacity to ‘live locally’, such as access to education, employment, social infrastructure, public open space, and transport related services and amenities, as well as housing affordability, and overall walkability and liveability (see Table 2, with further details in the Methods section).

**Table 2:** Description of the Urban Liveability Index and 13 core sub-indicators included in the published databases.

**Description (variable names listed in italics)**
Urban Liveability Index
The urban liveability index is a composite score based on performance across the 13 sub-indicators listed in this table^22,25^, and was calculated separately both for within-city (*uli_city*) and nationally (*uli_national*) relative comparisons of urban addresses and areas against a benchmark average score of 100.
Access to services and amenities
Access to destinations along the pedestrian road network for each address point was evaluated against destination specific access distance thresholds. A score out of 1 for access to destinations was calculated, using the soft threshold described by Higgs *et al*.^22^, and the average score for access to destinations within recommended thresholds was calculated using thematic categories, as listed below.
Social infrastructure; also see combined variable *social_infrastructure_mix* with score /16 for below destinations
1 Community, Culture & Leisure (*li_community_culture_leisure*): Community Centres (1000 m); Cinema/Theatre (3200 m); Libraries (1000 m); Museums/Art Galleries (3200 m)
2 Education access (*li_education*): State Primary Schools (1600 m); State Secondary Schools (1600 m)
3 Health & social services access (*li_health_services*): Aged Care (1000 m); Pharmacy (1000 m); Community Health Centres (1000 m); Dentists (1000 m); GP Clinics (1000 m); Maternal/Child Health (1000 m)
4 Sport & recreation access (*li_sport_rec*): Swimming Pools (1200 m); Sport/recreation facilities (1200 m)
5 Early years access (*li_early_years*): Childcare meeting quality requirement (any, 800 m; out of school hours, 1600 m)
Food
6 Fresh food access (*li_food*): Fruit/vegetable grocer (1000 m); Meat/seafood (3200 m); Supermarkets (1000 m)
Convenience
7 Convenience access (*li_convenience*): Convenience store (1000 m); Newsagent (3200 m); Petrol station (1000 m)
Transport
8 Access to regular public transport (*li_pt_regular_400m*) was evaluated using locations having average daytime (7 am to 7 pm) weekday service frequency of 30 minutes or less, considered across all public transport modes (e.g., bus, ferry, train, tram, as applicable) for stops during the Spring school term period of 8 October and 5 December.
Public Open Space
9 Access to large public open space (>1.5 hectares; *li_public_os_large_400m*) was evaluated using proxy entry point locations generated at 20 metre intervals along the boundaries of areas of open space located within 30 m of the walkable road network, and having publicly accessible area larger than 1.5 hectares.
Walkability; also see within-city (*walkability_city*) and between-city (*walkability national*) walkability index variables
10 Street connectivity per km^2^ (*li_street_connectivity_1600m*) was calculated as the number of pedestrian network intersections intersecting the local walkable network buffer, divided by its area in square kilometres.
11 Dwelling density per hectare (*li_dwelling_density_1600m*) was calculated as the sum of dwellings within Mesh Blocks (small statistical geography areas, equivalent to a street block) intersecting the local walkable network buffer, divided by its area in hectares.
Housing
12 Housing affordability stress (*li_sa1_30_40_housing_stress*) was evaluated as the proportion of low-income households (in the bottom 40% of the Australian income distribution) spending more than 30% of their income on housing costs. For inclusion in the ULI, this measure was reverse-scaled to represent ‘housing affordability’.
Employment
13 The percentage of employed persons working in the same Statistical Area 3 (broader catchment, SA3) as the local area (Statistical Area 1; SA1) in which they live (*li_sa1_sa3_local_employment*) was calculated as a measure of local employment opportunities.

**Fig. 1:**
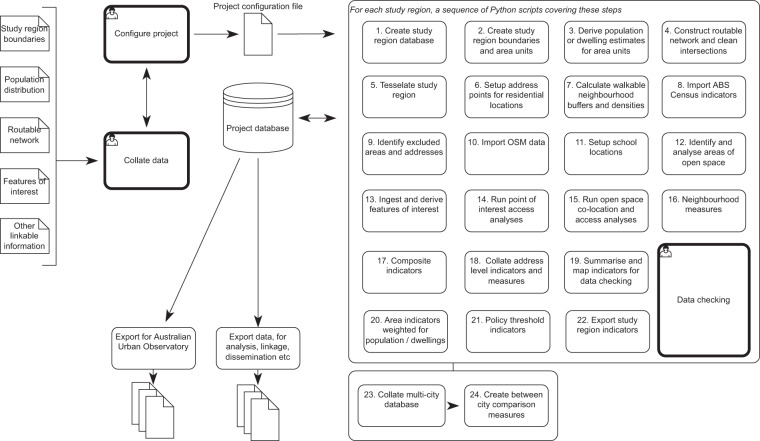
Scripted workflow for the calculation, validation, analysis, and dissemination of spatial urban indicator data prepared through the Australian National Liveability Study.

The project drew upon a Python-based scientific workflow (Fig. 1) developed to support the calculation of a policy-relevant and health-related composite indicator of urban liveability in Australia for urban residential locations: address points, and aggregate summaries for Australian Bureau of Statistics (ABS) Australian Statistical Geography Standard (ASGS) regions of Mesh Blocks (the smallest geographical unit for which census data is realised in Australia, with 30–60 dwellings and may be considered analogous to a street block) and larger Statistical Areas 1–4^19^, along with suburbs, Local Government Areas, and overall city summaries^19–21^. This approach was initially designed for calculation of a pilot urban liveability index (ULI) for Melbourne, Australia that was subsequently upscaled nationally^22^. The pilot ULI used 2011–12 data and allowed for flexible aggregation—from individual address point locations to larger area scales—for linkage, mapping and analyses as required to meet the needs of different stakeholders^22,23^. This enabled the workflow to be extended for the analysis of Australia’s state and territory capital cities in conjunction with Census data from 2016^5,24^, before further extension to regional areas for coverage of 21 cities with a target analysis time point of 2018.

## Methods

This study extended methods developed by Higgs *et al*.^22,25^ for calculating high resolution address level liveability indicators and an urban liveability index, supporting flexible aggregation to larger area scales. Figure 1 illustrates the Python-based workflow for built environment spatial and network accessibility analyses to construct a database of spatial indicators for Australian cities. The workflow encompasses indicator definition, data collation, project configuration, city-specific analyses, and compilation of a national database for export and dissemination. When describing specific indicators below, variable names for these indicators in the datasets are provided in italics. A summary of a series of core indicators is provided in Table 2. Full data dictionaries describing 196 indicators and measures for residential address points and progressively larger area aggregations (Mesh Block, Statistical Areas 1–4, suburbs, Local Government Areas, and overall city summaries) are provided in CSV and Excel format with the data and metadata files (see Table 1 and Data Records section).

### Indicator selection and definition

The study aimed to develop objective built environment measures for residential address points, corresponding to a range of indicators relating to a definition of ‘liveability’, that reflected the social determinants of health. Based on our team’s earlier program of work, liveable neighbourhoods were conceived as being “safe, attractive, socially cohesive and inclusive, and environmentally sustainable; with affordable and diverse housing linked by convenient public transport, walking and cycling infrastructure to employment, education, public open space, local shops, health and community services, and leisure and cultural opportunities”^11^. The term indicator is used to refer to a special kind of measure intended to be informative and ideally compared against some kind of agreed standard; these are measures designed to inform government officials and policymakers on progress towards achieving current policies, and provide evidence to inform strategies addressing specific issues^6,26^.

As noted above, the scope of the study spanned: (1) basic neighbourhood accessibility measures to several services and amenities; (2) indicators derived from these measures evaluating the degree to which thresholds recommended in policy and the social determinants of health literature have been met; and (3) composite measures for summarising the combined influence of neighbourhood characteristics. Examples of basic neighbourhood measures include the walking distance in metres (m) to the closest of a range of amenities, for example a supermarket (*dist_m_supermarket*). Using these built environment measures, indicators were derived for evaluating the extent to which urban policies were being achieved. For example, assessing whether each residential address meets the policy target of having an activity centre with a supermarket within 1000 m, or summarising an estimate of the percentage of dwellings in an area meeting this criterion (*walk_02*).

An example of a composite measure that directed the scope of this project is the walkability index. This is a relative measure of the degree to which local neighbourhoods support local living and active, sustainable lifestyles through the combined influence of dwelling density (*li_dwelling_density_1600m*), street connectivity (*li_street_connectivity_1600m*) and access to local amenities and services (*daily_living_access_1600m*). Traditionally, walkability indices incorporate a mixed-use entropy measure^27,28^. However, when undertaking our Australia-wide urban liveability study it was not possible to develop the mixed-use variable nationally due to unavailability of high-resolution land use data (e.g., retail floor area) with adequate coverage^9,29–31^. The daily living access indicator —a score out of 3 for access to a supermarket, public transport and convenience store within a 1600m walking distance— was therefore developed as an alternate approach to capturing mixed land use across a range of amenities and services^31^. A score for local access to several social infrastructure services and amenities was also developed (*social_infrastructure_mix*)^12^.

Small area summary indicators derived from ABS 2016 Census data^32^ were also linked with address points, representing characteristics of their broader neighbourhood (Statistical Area 1), including: the percentage of low-income households experiencing housing stress (spending more than 30% of income on rent or mortgage; *li_sa1_30_40_housing_stress*); and the percentage of employed persons with local employment (in the Statistical Area 3 catchment surrounding the address location’s Statistical Area 1; *li_sa1_sa3_local_employment*). Additional area level characteristics which may provide important covariates when conducting statistical analyses were also linked. This included area dwelling and person counts^7^, area size, and the ABS Statistical Area 1 Socio-Economic Indices for Areas (SEIFA) Index of Relative Socio-economic Disadvantage (IRSD)^33^.

**Fig. 2:**
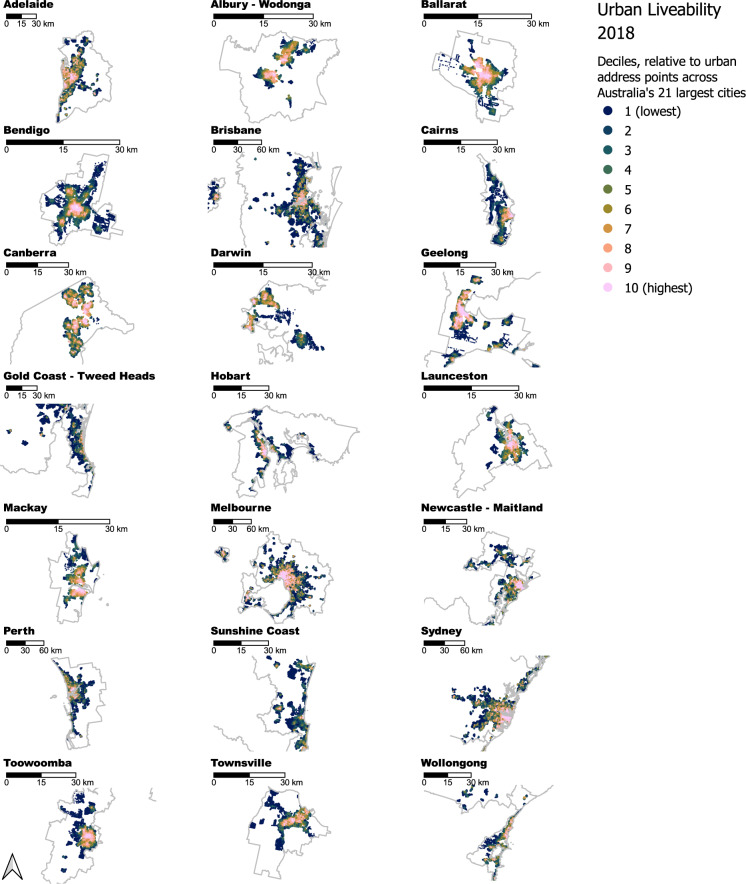
Spatial distribution of deciles of the urban liveability index calculated for residential address points across 21 of Australia’s most populous cities. For context, neighbouring cities have been retained in the sub-plot maps (e.g., Ballarat and Geelong are visible in Melbourne’s map, respectively to the west and southwest).

An overall urban liveability index (Table 2) was calculated as a composite of the other built environment indicators. It assessed the spatial distribution of liveability for the 21 cities included in the study (14 regional and seven capital cities), with both within-city (*uli_city*) and national (*uli_national*) standardisation. The measure was designed to help target interventions to address within- and between-city inequities (Fig. 2), and encourage exploration of the underlying determinants of liveable neighbourhoods using an interactive web map application (the prototype of which later became the Australian Urban Observatory).

The methods for construction of the urban liveability index and associations with health-related behaviours and outcomes have been published elsewhere^23,34^. Briefly, the urban liveability index is a variance-penalised average of 13 sub-indicators (Table 2) calculated for address points. In this way, the index is intended to implicitly reward consistency in performance across all domains. Sub-indicators with values exceeding 3-standard deviations of the mean are conditionally transformed to constrain the influence of those outliers, re-scaling observations in excess of 2-standard deviations to terminate within 3-standard deviations of the mean. Sub-indicators were normalised to have a mean of 100 and standard deviation of 10 prior to calculation of the urban liveability index, as per the formulation of the Mazziotta-Pareto Index upon which the composite measure was based^35^.

### Collating national data sources

To enable consistent assessment of urban liveability, data availability and comparability were major considerations when upscaling this research programme to include both capital cities and regional centres across jurisdictions nationally. Where possible, datasets with national coverage, consistent definitions, and target period of 2018 for features were prioritised; in particular, data of the ABS, and OpenStreetMap, as described below. Data sources used are detailed in Table 3.

**Table 3:** Data sources used in the 2018 Australian National Liveability Study.

Custodian	Year	Dataset and reference
Australian Bureau of Statistics	2016	ASGS Volume 1 geopackage boundaries (Mesh Block, Statistical Areas 1–4, Greater Capital City Statistical Areas)^19^
	2016	ASGS Volume 3 geopackage Suburb and LGA geometries^21^
	2016	ASGS Volume 4 geopackage (Significant Urban Areas, Urban Centres and Localities, Section of State)^20^
	2016	Mesh Block dwelling and person counts^7^
	2016	State Suburbs and Local Government Areas ASGS Edition 2016 in.csv Format (for Mesh Block linkage codes)^68^
	2016	SEIFA IRSD 2016 (SA1 Index of Relative Socio-economic Disadvantage)^33^
PSMA	2018	Geocoded National Address File (G-NAF) for Address locations, used in urban areas with dwellings^36^
OpenStreetMap		Points of interest (see Supplementary Table 1) and Areas of Open Space derived from OpenStreetMap 1 October 2018 planet dump file^40^
	2018	Pedestrian network and intersections generated using
		OSMnx from OpenStreetMap (via Overpass API http://overpass-api.de/)^39^
ACECQA	2019	Australian Children’s Education & Care Quality Authority (ACECQA) childcare centres (geocoded)^41^
ACARA	2019	Australian Curriculum, Assessment and Reporting Authority (ACARA), Primary and secondary schools, by sector (geocoded)^42^
Healthdirect Australia	2017	National Health Services Directory (accessed via AURIN Portal, https://portal.aurin.org.au/)^43^
Healthy Liveable Cities Lab	2016–2018	Additional geocoded datasets curated by Health Liveable Cities Lab from multiple sources: web-scraped supermarket and fast-food major chains, 2017^24^; Australian public lending libraries, 2016–18^44^
State Transport agencies	2018, 2019	GTFS feed data covering the period 8 October to 5 December for 2018 and 2019^69–78^

#### Census and boundary data

Data from the ABS 2016 Census was used extensively to characterise urban study regions: boundaries from the ASGS^19,20^ and related non-ASGS structures^21^; Mesh Block population and dwelling counts^7^; and Census data relating to employment and housing^32^. Data from the ABS was used under Creative Commons CC BY 4.0 licence terms.

#### Geoscape geocoded national address file (G-NAF)

Address locations across Australia were identified using the 2018 version of the PSMA (now Geoscape) Geocoded National Address File (G-NAF) dataset, released on the Australian Government’s national open data portal (data.gov.au) under a Creative Commons CC BY 3.0 licence^36^.

#### OpenStreetMap data

OpenStreetMap is a publicly accessible, collaborative mapping platform with global scope and an open data ethos. Launched in 2004, it has become an important source for consistently coded road network and features of interest data, including locations such as fresh food markets, convenience stores, and areas of open space. Completeness of coverage has been evaluated as being very high for urban areas with favourable comparisons to similar road and feature datasets^37,38^. There are established tools for using OpenStreetMap data in geospatial urban transport analysis, in particular OSMnx which this study drew upon to derive a pedestrian traversable network^39^. OpenStreetMap data is regularly archived, with usage predicated on the assumption that encoding of features provides an approximate representation of the real world at that point in time. Contributors to OpenStreetMap mark features using combinations of terms called key-value pairs, also known as tags.

The then-current OpenStreetMap planet database file was acquired on 1 October 2018 from https://planet.openstreetmap.org/pbf/planet-latest.osm.pbf, under the Open Data Commons Open Database License (ODbL)^40^. Our study drew upon OpenStreetMap TagInfo (https://taginfo.openstreetmap.org/)—a tool for exploring how features have been represented (or tagged) in OpenStreetMap—to conduct an audit identifying the most frequently used tags to represent points of interest (POIs) for our study in November 2018. These were tags were used, in addition to established guidelines for tagging destinations in OpenStreetMap, for specific types of destinations of interest to our study (Table 4). In addition to informing a derived pedestrian road network, OpenStreetMap was used to represent POIs and areas of public open space where official data with national consistency and coverage were not otherwise available.

**Table 4:** Example guidelines for tagging of amenities.

Concept	Tagging guidelines
Australian Tagging Guidelines	https://wiki.openstreetmap.org/wiki/Australian_Tagging_Guidelines
Supermarkets	https://en.wikipedia.org/wiki/Supermarket
Markets	https://wiki.openstreetmap.org/wiki/Tag:amenity%3Dmarketplace
Shops	https://wiki.openstreetmap.org/wiki/Key:shop
Convenience stores	https://wiki.openstreetmap.org/wiki/Tag:shop%3Dconvenience
Public open space	
*Green space*	https://wiki.openstreetmap.org/wiki/Green_space_access_ITO_map
*Public squares*	https://wiki.openstreetmap.org/wiki/Tag:place%3Dsquare
*Other kinds of public areas designed for or used by pedestrians*	https://wiki.openstreetmap.org/wiki/Tag:highway%3Dpedestrian

POIs represented using OpenStreetMap included fresh food and supermarket outlets, convenience stores, community centres, cultural institutions and public swimming pools. However, as a contribution to the literature we include a full set of terms identified through our audit that includes additional categories not analysed in our final set of indicators (Supplementary Table 1). When conducting ‘distance to closest’ analysis some destinations were pooled with other data sources to reduce risk of error. For example, distance to closest supermarket also considered access to major chain supermarkets retrieved from web-scraping. Further detail on tags used and the approach taken when using OpenStreetMap data is provided in the Technical Validation section.

#### Generalised transit feed schedule data

State and territory public transport agency Generalised Transit Feed Schedule (GTFS) data under the Creative Commons CC BY 4.0 licence were used to determine location and frequency of service of public transport across available modes nationally (Table 3). These locations were used to evaluate access to public transport meeting specific service frequency criteria.

#### Additional data sources

Additional specialty data sources acquired for processing specific indicators and points of interest access measures are summarised in Table 3, including data from: the Australian Children’s Education & Care Quality Authority (ACECQA)^41^; Australian Curriculum, Assessment and Reporting Authority (ACARA)^42^; National Health Services Directory^43^; and additional geocoded datasets curated by Health Liveable Cities Lab at RMIT University, Melbourne, Australia from multiple sources (major supermarket and fast-food chain locations^24^; and public lending library locations^44^).

### Project configuration

Project settings were defined across sheets in an Excel workbook (Table 5), which allowed analysts with limited programming experience to readily engage with and customise the Python-based scripted workflow (Fig. 1). The fields used to define indicators in the configuration file are described in Table 6. Analyses were programmed in Python 2.7^45^, employing a PostgreSQL 12.2 database server with the PostGIS 3.0 spatial analysis extension^46,47^. OSGeo4Win ogr2ogr GDAL 3.0.4 was used for ingestion and conversion of spatial data between different formats^48^. The OSMnx Python module was used for deriving a walkable pedestrian network using a custom walk/cycle definition^39^. Network analyses were undertaken using the ArcGIS 10.6 Network Analyst extension, via the arcpy Python module^49^. QGIS 3.10.1 was used for visualisation and data-checking through the course of analyses^50^, along with psql 12.2 for database querying^46^. Spatial analyses for all cities were undertaken using the GDA2020 Geoscience Australia Lambert Conic Conformal projection (GDA2020 GA LCC, EPSG 7845), with spatial transformations undertaken using NTv2 transformation grids.

**Table 5:** Project configuration worksheet summary.

Worksheet	Purpose
parameters	Establishes the key parameters for a project using this set of scripts (e.g., spatial reference, year, buffer sizes, etc)
regions	Used in scripts to define data sources and parameters for geographic areas and geographic linkage information (rows)
study_regions	Study region definitions
destinations	Location and classification of destination points of interest
indicator_setup	Indicator definitions
observatory	Variables intended for aggregation, export and subsequent dissemination via the Australian Urban Observatory
data_catalogue	Catalogue of data sources used in the project and associated metadata
osm_dest_definitions	OpenStreetMap destination classification
osm_and_open_space_defs	OpenStreetMap definitions to identify areas of open space
nhsd classification	Classification of National Health Service Directory (NHSD) service categories to destination types
aedc	Variables required for the AEDC linkage project, linkage parameters, and the naming schema for these
ULI	A record of destinations included in the Urban Liveability Index and how these map to specific domains and other indicators

**Table 6:** Configuration fields used to define liveability indicators in the ‘indicator_setup’ worksheet of the configuration file.

Field	Description
indicators	Short variable name for indicator, with domain grouping
domain	A conceptual domain under which the indicators have been categorised
scale	The geographic scale of the indicator
ind_plain	A slightly longer plain English name for a variable, where possible
tags	Permutations of indicators using soft or hard thresholding^22^
locale	The study region to which this indicator relates (for national study, this is an asterisk indicating all 21 cities)
ntnl_scripts	Whether the indicator has been incorporated into the scripted workflow
updated?	The date that this indicator was last incorporated or updated in the scripted workflow
unit_level_description	Description of this indicator as it relates to address points
aggregate_description	Description of this indicator as it relates to areas
threshold_aggregate_description	Description of this indicator as it relates to areas with evaluation as a percentage based on a threshold split
notes	Any notes about this indicator
policy_locale	Whether this indicator relates to policies in specific study regions
policy_wording	Wording of any policy reference for this indicator
policy_reference	The policy reference for this indicator
data_sources	A shorthand summary of data sources used, where completed
Query	The final main SQL query used to construct this indicator from intermediary tables as part of the scripted workflow
Source	Linkage query portion for the source intermediary SQL database tables used to construct this indicator
agg_form	The form of this indicator once aggregated (e.g., an average, percentage, ratio or Z-score)
agg_scale	Any scaling to be applied to aggregate indicators (e.g., multiplying a proportion by 100 to represent a percentage)
agg_alt_variable	Whether the aggregate statistic is calculated using an alternate table to the address level indicator (which may not exist in some cases)
agg_standard	Whether the indicator is to have weights applied on aggregation, e.g., to represent dwellings or persons
agg_split_greq	A split point for evaluating thresholds meeting a certain value
units	Units of the indicator (can include html for web map descriptions of area level indicators)
polarity	Polarity (e.g., Ascending or Descending)

### City-specific analyses

City-specific analyses were divided amongst three analysts (CH, JR and RR) and performed on Alienware Area-51 R2 x64 computers each with i7 3.2Ghz 8-core processors and 32GB ram. Study region specific parameters were used to run a sequence of scripted analyses for each city^22^, as detailed in Fig. 1. The methods expand on those described previously by Higgs *et al*.^23^, with additional scope for Australia’s 21 largest cities, use of OpenStreetMap data, and use of GTFS data which was not available for the earlier pilot analysis targeting 2012.

After initialising the city database, study region boundaries were imported and linked with data on dwellings and population. Address points located in Mesh Blocks with dwellings were identified, along with environmental features within 10 km of the urban study region boundary. These included a derived pedestrian road network and intersection dataset (see Technical Validation section), POIs and areas of open space (see Technical Validation and Supplementary Material sections). The additional 10 km buffered distance was used to mitigate the risk of edge effects for peri-urban addresses, who may be able to access services and amenities outside the identified city boundary. Local neighbourhood analyses were conducted, and then used to derive indicators and composite indices for address points. More detail on each of these steps is provided below, and in the Technical Validation and Supplementary sections.

Urban study regions for the 21 cities were defined using ABS ASGS boundary geometries as the intersection of urban (Major- or Other-) Sections of State with either Greater Capital City Statistical areas (for state and territory capital cities) or Significant Urban Areas (for regional cities)^19,20^. City-specific extracts were processed using.poly boundary files generated based on the 10 km buffered geometry of each urban study region. The scripted analysis process for each study region involved ingesting the pre-extracted corresponding OpenStreetMap portion using osm2pgsql (https://github.com/openstreetmap/osm2pgsql) into the study region database as line, point and polygon features. The tags used to define OpenStreetMap destinations were defined in the project configuration file, and are summarised in Supplementary Table 1.

Residential address points across Australia’s 21 largest cities were identified, located within urban Mesh Block small areas with positive dwelling counts from the ABS 2016 Census^7,36^. These points served as proxy locations for urban residences for which a suite of planning and policy relevant built environment distance and density measures and indicators were derived. Indicator estimates were calculated for 6,888,547 address points (including some in non-urban locations) across the 21 cities using OpenStreetMap and other data sources with a target year of 2018 for pedestrian network analyses and spatial-relations analyses. Local neighbourhoods were analysed identifying the walkable catchments around residential address points, and calculating the corresponding dwelling and intersection density statistics for these. Network analysis for the distance to closest destination was conducted for all destination categories, in addition to calculating the distance to all destinations of each category within 3200 m (a value relevant to walkability policy being twice the walkable catchment of 1600 m). The latter measure resulted in distance arrays which could be queried to return the count of destinations within policy relevant distances, as required (e.g., count of fresh food outlets within 400 m, 800 m, 1600m and 3200 m), supporting post hoc querying (see Data Records). Such ‘count within distance’ analyses were only conducted with destinations sourced from a single origin, to ensure no double counting occurred (see discussion of pooling data in ‘Evaluating access to closest supermarket’ in Technical Validation). In a similar way, the unique ID and distance of all public transport stops within 800 m, and the closest, were recorded, supporting the calculation of a series of policy relevant public transport indicators for address points. The co-location of destinations within 100 m of areas of open space was recorded, as part of the detailed set up of the areas of open space dataset which supported analyses of a variety of public and other open space typologies (see Technical Validation and Supplementary Material sections). To evaluate access to open space, the unique ID and distance to closest entry point for all areas of open space within 3200 m was recorded.

Indicators for residential address locations were then derived using the calculated measures. The validity of the core neighbourhood measures was evaluated; address points were flagged for exclusion in the final data if they did not meet the urban residential inclusion criteria (331,895 records; 4.8%), or due to identification of invalid network topology (n = 20,252; 0.3%). The latter could arise when an address was matched to a network segment disconnected from the main network, resulting in invalid results of local walkable neighbourhood analyses (Table 7). Composite indicators including the walkability index, social infrastructure mix score, and urban liveability index (see Table 2) were constructed. The resulting dataset provided a rich spatial census of the built environment for 6,536,400 records, supporting aggregation at a range of larger scales including Statistical Areas, suburbs and local government areas with weighting for Mesh Block persons and dwellings.

**Table 7:** Summary counts of residential address points excluded and included in the final dataset.

Exclusion and inclusion groups	Count	%
Unique address locations across 21 cities prior to exclusions	6,888,547	100.00
Does not mean inclusion criteria	331,895	4.82
*not urban*	*312,006*	*4.53*
*not in an SA1 with SEIFA IRSD*	*19,889*	*0.29*
Invalid network topology	20,252	0.29
*50 m buffered 1600m network area less than plausible minimum of 16.5 hectares*	*17,993*	*0.26*
*Other connectivity issues resulting in null results for walkable neighbourhood measures*	*2,259*	*0.03*
**Final set of residential locations included (not excluded)**	**6,536,400**	**94.89**

### National database, aggregation, export and dissemination

Core indicator results were exported from the city-specific databases and collated to form a combined 21 city ‘Australian’ database of data and measures across all study regions. Further analysis was conducted to support between-city comparisons; specifically, a national walkability index and a national liveability index. Table 2 describes the Urban Liveability Index and its 13 core sub-indicators. Further descriptions are provided as supplementary material.

Address point measures were linked with geocoded survey data to support analysis examining associations with individual health and wellbeing outcomes (e.g., the Australian Early Development Census^51^). Small area and larger aggregate summary measures were used to evaluate the implementation of policies and identify inequities within cities. These were mapped at different scales (neighbourhood, local government area and city-wide) and disseminated to policymakers and practitioners through the Australian Urban Observatory^34^, along with liveability reports for 21 Australian cities^52^. City summaries were provided as high-level liveability indicators and included in the Australian Government’s National Cities Performance Framework^53^. Projects making use of this data are summarised elsewhere, along with lessons learnt from the project’s scaling up^8^.

## Data Records

The Australian National Liveability Study 2018 indicators and measures have been consolidated across 14 distinct datasets each in plain text (CSV) format for archival purposes (Table 1), published on RMIT University’s Figshare repository^54^. The datasets are stored along with 7 corresponding machine readable (CSV) data dictionaries detailing the dataset variables, their description and data type (cross-referenced listings in Table 1), a machine-readable (CSV) file cross-referencing datasets and data dictionaries (0 - Datasets and Data Dictionaries.csv; corresponding to Table 1), a formatted Excel workbook (XLSX) version of the data dictionaries including hyperlinked cross-references for each dataset file with its description (Australian National Liveability Study 2018 - Data Dictionaries.xlsx; corresponding to Table 1), machine readable (CSV) descriptions of data sources (8 - Data sources.csv; corresponding to Table 3), machine readable (CSV) descriptions of OpenStreetMap destination definitions used (9 - OSM Destination Definitions.csv; corresponding to Supplementary Table 1), a machine readable (YAML) metadata file (*metadata.yml*), and a JPEG reproduction of Fig. 2 as an applied example of the data’s usage (National Urban Liveability Index - 2018.jpeg). In addition, there is a 100-record sample of the ‘od_aos_jsonb’ TSV file to allow users to experiment with its usage without committing to load the full data contained in that particular dataset (*hlc_ntnl_liveability_2018_od_aos_jsonb_100_record_sample.tsv;* 265.1 kB, compared with 17 Gb for the full file described in Table 1). In total, there are 29 files published in the repository: the 14 datasets described in Table 1; 10 supplementary machine-readable CSV files (including data dictionaries cross-referenced in Table 1); 1 formatted Excel workbook cross-referencing datasets with data dictionaries; 1 metadata file; 1 example map; and 1 sample file recording areas of open space reachable within 3200 m for 100 randomly selected address locations from the full ‘od_aos_jsonb’ dataset.

The core indicator dataset is comprised of 6,536,400 records for urban residential address locations across Australia’s 21 largest cities (5.1 GB), with easting and northing coordinates recorded using GDA2020/GA LCC spatial reference (EPSG 7845) and a comprehensive set of area linkage attributes (Mesh Block, Statistical Area Levels 1–4, Suburb, LGA and city). These indicators have also been aggregated as averages for Mesh Blocks (183,075 records; 427 MB), and at seven larger area scales including overall city summaries, with weighting for Mesh Block dwelling counts. Users may replicate person-weighted indicator data by using the Mesh Block indicator dataset person counts to take weighted averages of indicators for the desired area scales (see Usage Notes). The address level dataset itself may also be used to support aggregation and linkage as required.

In addition to the core liveability indicator datasets, additional datasets are provided for residential address locations containing (1) estimates for distance in metres along pedestrian network to the closest of a range of destinations (3.9 GB), as well as (2) arrays of estimates for distance in metres along pedestrian network to all destinations (within 3200 m and the closest) across a range of destination types (11.6 GB). This latter dataset can allow for post hoc creation of count-based indicators for specific destinations, by evaluating the number of recorded distances that are within a specific threshold distance. For our analysis, we generated this data in a PostgreSQL database, with distance records stored as integer arrays (Integer[] datatype). A sample dataset of distance arrays for 100 residential address records has also been provided to allow users to experiment without loading the full dataset.

In addition to the indicator and built environment measure datasets, we also provided further derived datasets of destinations. A dataset of GTFS transport stops containing a headway analysis of day time weekday public transport service frequency (8 October 2019 to 5 December 2019) with Well Known Text (WKT) geometry has been provided in CSV format, using data sources described in Table 3 and analysed using R with Tidy Transit^55^. A richly attributed dataset of publicly accessible areas of open space has also been provided in zipped CSV format (19.4 GB unzipped; 1.94 GB zipped) containing WKT geometries (overall area, public area, water area), jointly indexed by study region and a sequential identifier. In conjunction with a further dataset detailing distances and identifiers for each residential address within 3200 m, this allows for post hoc querying on distances to public open space meeting specific attributes (e.g., of a particular size, co-location within 100 m of a public toilet or other amenities, having a water feature, etc.). Details on datasets are provided in Table 1. Further details on using and querying these datasets is provided below in the Usage Notes section.

## Technical Validation

To facilitate meaningful comparisons of local neighbourhood attributes for addresses and areas across diverse cities around Australia, the 2018 Australian National Liveability Study required identification of datasets with broad coverage and consistent definitions across jurisdictions. As described above, OpenStreetMap was utilised as a nationally consistent open data source for pedestrian routable roads and walking path data, as well as for a range of points of interest. Others have previously demonstrated validity of the use of OpenStreetMap for developed urban areas such as those included in this study^38^. However, to examine these assumptions, through the course of our study we conducted validation experiments and sensitivity analyses, including investigation of null and outlying values, ground truthing comparisons using satellite data, and systematic comparisons of OpenStreetMap derived features with those from official or commercial datasets, as described below.

### Approach to measuring and aggregating residential address point exposures

**Fig. 3:**
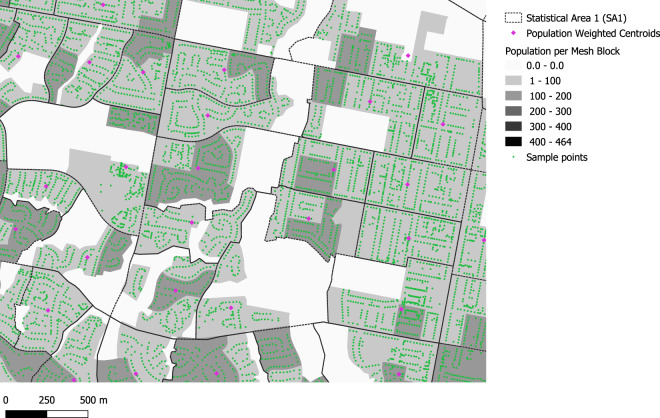
Comparison of SA1 population weighted centroids with residential address locations, and Mesh Block population counts in Wodonga, Victoria (Albury – Wodonga).

An early methodological decision in the project was to measure liveability indicators for residential address locations^23^, rather than approximate this using population weighted centroids as had been done in earlier work^56^. Address point data allowed for the disaggregated and aggregated data to be used for different purposes (i.e., linkage). For example, linkage of residential address indicator data to geocoded participant locations in health and other surveys allowed study of associations with health outcomes. Further, this also allowed measures recorded for these locations within Mesh Blocks with known residential dwellings to be readily aggregated to larger scales. Mesh Blocks with dwellings capture a two-dimensional spread of the locations where people may live, and small area counts of dwellings or persons from Census data could be used when aggregating to represent the average experience of persons or dwellings with regard to specific phenomena at a range of scales, while retaining the capacity to interrogate variation^57^. While a population-weighted centroid aims to capture an average location representative of experiences for a broader area, the risk is that this may result in measurement for the average location where nobody lives. This can be seen in Fig. 3, which contrasts population weighted centroids for SA1 areas with address points^36^ overlaid by population counts^7^, The population weighted centroid for the SA1 in the upper right corner is located in a location without any population count at the 2016 Census, and reliance on this single point risks mis-representing the average experience in that neighbourhood. In contrast, measurement using address points in Mesh Blocks with dwellings ensures a degree of robustness when aggregating upwards. While measurements for a single point may be an outlier in terms of neighbourhood representation, the average of a suite of points will provide a fairer representation of the ‘average experience’ for persons or dwellings, particularly when weighted for in the process of aggregation.

### Pedestrian network model

Evaluation of local neighbourhood walkability and access to amenities was underpinned by assumptions of valid street network data. Prior to commencement of the national analysis, in order to evaluate suitability of using OpenStreetMap data for accessibility analyses, we conducted preliminary investigations of these assumptions, which led to a refinement in our approach. We conducted a sensitivity analysis comparing results from usage of a pedestrian network derived from OpenStreetMap (June 2018) using OSMnx, with results arising from usage of an analogous pedestrian network previously derived from the 2013 Public Sector Mapping Agency (PSMA) Transport and Topography Street Network^58^, which excluded heavy roads and those inaccessible to pedestrians^29^. The OpenStreetMap-derived network was constructed using a custom pedestrian network filter based on the OSMnx ‘walk’ network type, omitting the exclusion of cycling (Function 1). This was considered desirable for modelling pedestrian accessible routes in the Australian context, where OpenStreetMap paths tagged for cycling were found to provide important connectivity traversable by pedestrians, for example across the Yarra river in Melbourne, which at the time were absent in the ‘walk’ network otherwise intended for walking behaviour.

Function 1. Custom pedestrian network function used to construct the OpenStreetMap-derived routable pedestrian network.

pedestrian = (‘[“area”!~”yes”][“highway”!~”motor|proposed|construction|abandoned|platform|raceway”][“foot”!~”no”][“service”!~”private”][“access”!~”private”]’)

Using each of these derived pedestrian network datasets, we conducted a preliminary network analysis of the distance to closest bus stop (2012 data) for residential address points with unique locations within the Melbourne ABS 2016 Greater Capital City Statistical Area (GCCSA) using road networks and destinations extending to 10 km beyond the GCCSA boundary. Restricted to the ‘Major Urban’ or ‘Other Urban’ Sections of State, there were 1,718,271 residential address points in the urban portion of Greater Melbourne. Origin-Destination matrix (OD matrix) analyses were conducted using 64-bit Python 2.7 with the ArcGIS arcpy library and Network Analyst extension with results output to an SQL database using PostgreSQL 9.6. We examined differences in the distribution of distance to reach a bus stop and the overall count of null values using each network type. A null result was interpreted as being suggestive of isolated failures to represent real world network connectivity in this urban context where access to a bus stop within a reasonable distance could be expected for most address point origins. The results using the PSMA-derived network returned 2,083 nulls (0.12%), whilst those using the preliminary OpenStreetMap derived network returned 40 (0.002%). The additional modest number of null values in the PSMA network may be partially accounted for by the difference in date of network data publication (2013, compared to 2018); most real-world network changes would be expected to occur in new developments on the urban fringe. To facilitate fair comparisons of differences in distributions, summaries were conducted only for address points with observations in common using both network sources (n = 1,716,150).

For each address point, the distance to closest bus stop calculated using the OpenStreetMap-derived network was subtracted from the results arising from use of the PSMA 2013 network, where findings were returned for both networks. The resulting differences provide an indication of similarity, as summarised in Table 8. The difference for most addresses was less than 10 m (interquartile range −3 to 8 m), while the median difference was 1 m. These differences were positively skewed, indicating that analysis using the OpenStreetMap derived network resulted in distance estimates that for most addresses were shorter than were the PSMA network used, reflective of a greater connectivity. While most differences were on average small (11 m) some were large enough to be meaningful (standard deviation of 168 m). In outlying circumstances some addresses would travel more than 500 m further using the PSMA network to reach a bus stop (99^th^ percentile of difference), while using the preliminary OpenStreetMap-derived network the outlying scenario approached 500 m.

**Table 8:** Summary of distribution of distances (m) to closest bus stop undertaking using official PSMA road network and a preliminary OpenStreetMap-derived network and that used in the final analysis, as well as the difference in results using these (pooling exclusions for missing records for fair comparison of shared distributions).

Pedestrian network comparison analysis	N *(excludes missing)*	Missing	Percentile					Mean	SD
			1	25	50	75	99		
**Distance to closest bus stop (2012); distances exclude nulls for both datasets (i.e.the combined missing)**									
*PSMA network (reference)*	1,716,150	2,083	8	187	331	550	3,025	490	745
*OpenStreetMap-derived (June 2018)*	1,716,150	40	10	187	326	536	2,871	479	738
*Difference (PSMA – OpenStreetMap)*	1,716,150	2,121	−348	−3	1	8	503	11	168
**Distance to closest bus stop (2012 vs 2018); distances exclude nulls for both datasets and exclusions described in Table** 8)									
*PSMA network (reference)*	1,711,880	2,083	8	187	331	549	3024	490	745
*OpenStreetMap-derived (October 2018)*	1,711,880	4,322	10	184	317	504	1,980	423	582
*Difference (PSMA – OpenStreetMap)*	1,711,863	6,408	−494	−4	1	13	1,790	66	530
**Distance to closest bus stop (2012); distances exclude null for both datasets, and exclusions described in Table** 8)									
*PSMA network (reference)*	1,711,863	2,083	8	187	331	549	3,024	490	745
*OpenStreetMap-derived (June 2018)*	1,711,863	40	10	187	326	536	2,858	479	738
*Difference (PSMA – OpenStreetMap)*	1,711,863	6,425	−344	−3	1	8	499	11	167

When conducting a post hoc comparison analysis of results using the PSMA 2013 network and the final derived OpenStreetMap pedestrian network (October 2018) with the exclusions listed in Table 8, the distance to closest bus stop was found to be 66 m closer when using the approach adopted in the study, albeit with considerably variability (standard deviation 530 m). An important caveat with this comparison is that the preliminary analysis was conducted using bus stops from 2012 while the final analysis was conducted using bus stops from 2018, with some changes to locations of bus stops between those time points. This impacts comparability because in such cases the change in distance relates not to improved representation of pedestrian routing options or restriction to valid locations, but rather to change in the representation of where bus stops are located. For a fairer representation of the impact of the exclusions employed in the final analysis, comparison of the preliminary analysis using 2012 bus stops for both networks was repeated with these additional records excluded (n = 1,711,863). Little difference was observed, with most distributional estimates remaining unchanged from the analysis with 1,716,150 records.

### Length of road network by sections of state comparison

We also compared total road length by section of state classification (Major Urban, Other Urban, Bounded Locality, or Rural Balance) using the OpenStreetMap-derived pedestrian network (October 2018) and the official Victorian Vicmap roads 2018 dataset^59^, excluding freeways, proposed roads and boat/ferry routes. While the results were influenced by both the coverage and density of network representation, this overall comparison emphasises the strength of the OpenStreetMap-derived network for urban areas, and underscored the importance of restricting our liveability indicator analyses drawing on OpenStreetMap data to urban areas (Table 9).

**Table 9:** Total length of pedestrian road network paths extending 10 km beyond the Melbourne Greater Capital City Statistical Area, stratified by section of state, by road network source.

Section of State (SOS)	OSM-derived (2018)		Vicmap-derived (2018)	
	m	%	m	%
Major Urban	32,863,120	71.9	26,845,675	61.4
Rural Balance	10,282,032	22.5	14,485,760	33.1
Other Urban	2,172,592	4.8	1,939,091	4.4
Bounded Locality	400,811	0.9	429,217	1.0
Total	45,718,555	100.0	43,699,743	100.0

### Street intersection model

**Fig. 4:**
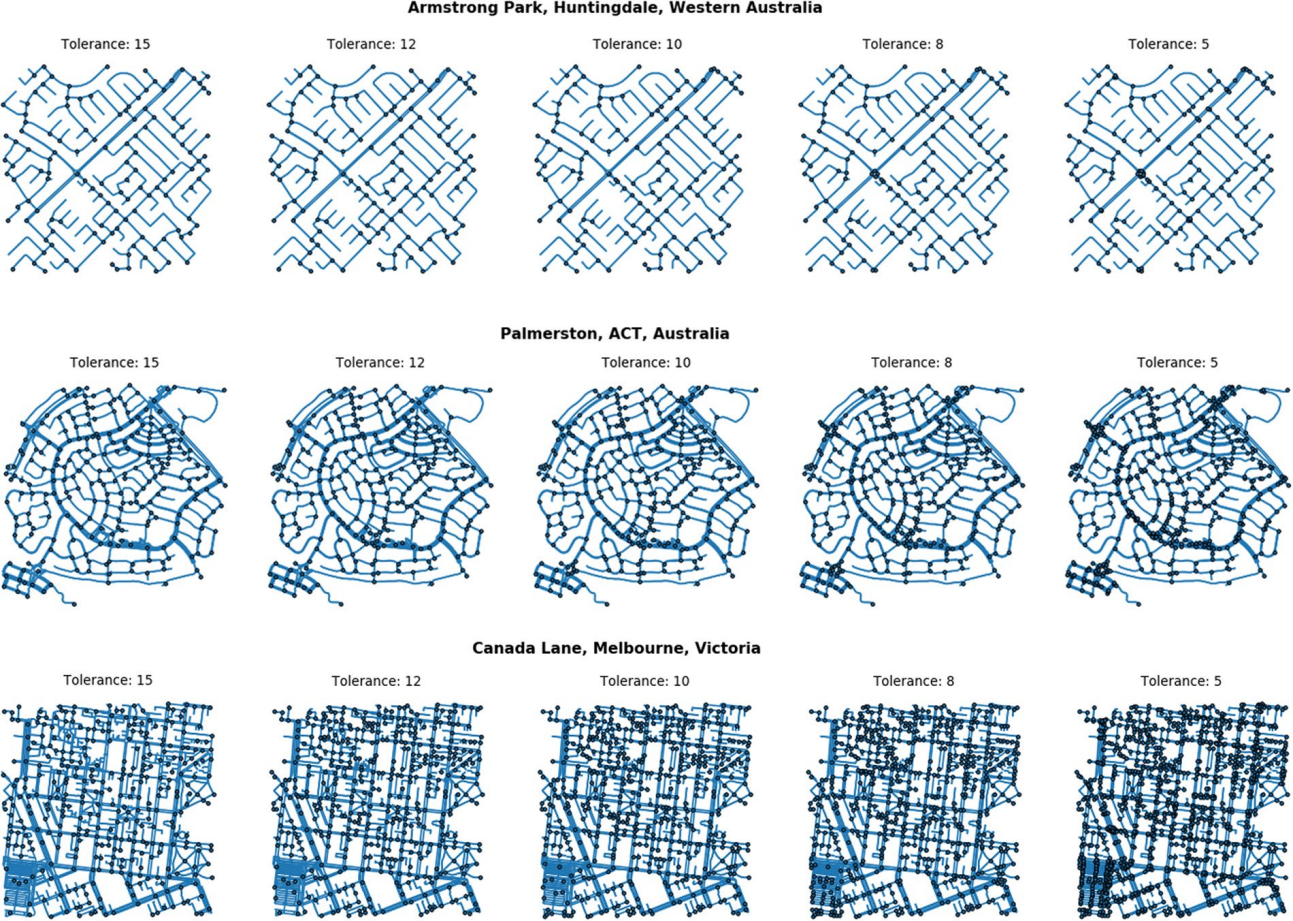
Examples of sensitivity analysis using 1500 m2 samples of different network topologies in different cities, evaluating use of cleaning tolerance of 5, 8, 10, 12, or 15 metres.

To evaluate street connectivity (e.g., intersections per km^2^), data containing representations of street networks for mapping or routing purposes required simplification of the intersections (nodes) of network segments (edges). For example, a mapped representation of a roundabout or large street intersection on OpenStreetMap may involve multiple points where lanes of traffic or other paths intersect. While this will not necessarily pose a problem for evaluating routing through the network (other than increased processing and memory demands arising from complexity), if those nodes are naively taken to represent real-world intersections, then measures of street connectivity for that location will be over-estimated. The OSMnx python module includes a function to simplify network topology, which given a parameter for tolerance distance will return the centroid of points identified within that spatial window^39^. For OSMnx 0.81 as used in the study, the function was clean_intersections(graph, tolerance, dead_ends = False); in more recent versions the equivalent function is consolidate_intersections(graph, tolerance, rebuild_graph = False, dead_ends = False). We conducted a sensitivity analysis to evaluate the choice of parameter across different network topologies identified in different Australian cities, for example residential neighbourhoods with roundabouts and cul-de-sacs in Perth and Canberra, and an area of Melbourne’s CBD with tight laneways and market areas (Fig. 4). Based on this analysis, we determined that to approximate the cleaning algorithm used in our previous work for the common network topologies observed in Australian cities using the October 2018 export of OpenStreetMap, a tolerance distance of 12 m was an appropriate compromise for these settings.

### Modelling and evaluating access using public open space

Public open space means different things to different people, and in the context of this national study we sought a consistent definition which we applied across jurisdictions. The Victorian Planning Authority defines open space as land providing outdoor recreation, leisure and/or environmental benefits and/or visual amenity; and public open space as land which is publicly owned, accessible, has primary purpose for outdoor recreation, leisure conservation, waterways and/or heritage, and meets the definition of open space^60^. This definition that we attempted to approximate using data derived from OpenStreetMap may describe a broad range of public places, including parks, squares, beaches, and conservation areas. This approach is further detailed in the supplementary material usage notes located at https://github.com/carlhiggs/Australian-National-Liveability-Study-2018-datasets-supplementary-material.

To evaluate the impact of choice of public open dataset on estimates of dwelling with access to public open space, we conducted a preliminary analysis comparing estimates for percentage of dwellings having access to a public open space within 400 m meeting a series of conditions: (1) any public open space; (2) having area of 1 hectare or larger; and (3) having area of 1 hectare or larger, or any size with a sports facility. Evaluation of access to any public open space was based on a measure used by the VPA^60^. Evaluation of the latter two typologies was based on Standard C13 of the Victorian Planning Provisions^61^: ‘*Local parks within 400 metres safe walking distance of at least 95 percent of all dwellings. Where not designed to include active open space, local parks should be generally 1 hectare in area and suitably dimensioned and designed to provide for their intended use and to allow easy adaptation in response to changing community preferences*’. Public open space feature datasets were derived using two official Victorian open space datasets—Victorian Planning Authority (VPA) and Vicmap Features of Interest (FOI)—as well as OpenStreetMap retrieved for a 10 km expanse beyond the boundary for Greater Melbourne. The OpenStreetMap-derived datasets (preliminary versions 1 and 2, and the final one we employed in our study) were constructed using a series of tags informed by review of the VPA definitions, OpenStreetMap tagging guidelines for public open spaces, and empirical review of satellite data for the included cities in our study. Access was further evaluated using both the OpenStreetMap-derived and Vicmap-derived pedestrian networks based on 2018 data. The VPA open space dataset used for comparison analysis was created in 2016 as part of a review into Melbourne’s metropolitan open space network and included open space features pre-categorised into Public, Restricted or Private open space; while we considered this a gold standard reference for public open space data, it had coverage only for 32 municipalities of Greater Melbourne (missing Murrindindi Shire, Mitchell Shire, Macedon Ranges Shire, Moorabool Shire)^60^. The Vicmap FOI open space dataset was created by the Victorian Government Department of Environment, Land, Water and Planning in 2016 and last updated prior to our retrieval in 2018; it is available as part of Vicmap’s Features of Interest dataset, with state-wide coverage and planned annual update subject to available funding^62^. Results of this analysis are presented in Table 10.

**Table 10:** Comparison of estimates for dwellings with access to public open space (any; ≥1 hectare; ≥1 hectare or having a sport facility), by derived pedestrian road network (OpenStreetMap or Vicmap Transport) and source of public open space data.

	Any public open space				Public open space of area ≥1 hectare				Public open space ≥1 Hectare, or any size having a sport facility			
	Major Urban	Other Urban	Rural Balance	Bounded Locality	Major Urban	Other Urban	Rural Balance	Bounded Locality	Major Urban	Other Urban	Rural Balance	Bounded Locality
**Preliminary analysis using public open space datasets derived from OpenStreetMap, VPA (gold standard) and Vicmap FOI**												
**Victorian Planning Authority (gold standard)**												
*Using OpenStreetMap network*	91.4	69.2	32.5	61.9	57.1	49.3	22.5	44.1	57.9	50.0	23.1	45.2
*Using Vicmap Transport network*	91.8	69.8	32.7	62.9	57.2	49.9	22.9	45.3	58.0	50.5	23.5	46.9
*Difference in network results*	0.4	0.6	0.2	1.0	0.1	0.6	0.4	1.2	0.1	0.5	0.4	1.7
*Difference to gold standard*	—	—	—	—	—	—	—	—	—	—	—	—
**Vicmap FOI**												
*Using OpenStreetMap network*	84.8	83.0	28.2	59.5	56.6	53.4	18.9	37.0	58.6	54.0	19.2	37.8
*Using Vicmap Transport network*	84.9	83.8	28.8	62.9	56.6	53.6	19.2	40.4	58.5	54.2	19.6	41.7
*Difference in network results*	0.1	0.8	0.6	3.4	0.0	0.2	0.3	3.4	−0.1	0.2	0.4	3.9
*Difference to gold standard*	6.6	−13.8	4.3	2.4	0.5	−4.1	3.6	7.1	−0.7	−4.0	3.9	7.4
**OpenStreetMap (version 1)**												
*Using OpenStreetMap network*	84.8	67.2	40.4	58.2	59.7	50.0	35.3	50.7	63.1	51.0	35.6	51.6
*Using Vicmap Transport network*	84.9	67.6	42.1	59.9	59.5	50.5	36.9	54.5	62.8	51.3	37.1	55.0
*Difference in network results*	0.1	0.4	1.7	1.7	−0.2	0.5	1.6	3.8	−0.3	0.3	1.5	3.4
*Difference to gold standard*	6.6	2.0	−7.9	3.7	−2.6	−0.7	−12.8	−6.6	−5.2	−1.0	−12.5	−6.4
**OpenStreetMap (version 2)**												
*Using OpenStreetMap network*	84.7	67.4	36.3	56.9	53.3	41.1	12.4	29.6	55.8	42.0	13.1	31.6
*Using Vicmap Transport network*	84.7	67.7	38.0	58.4	53.0	41.1	12.8	31.0	55.5	41.9	13.5	32.2
*Difference in network results*	0.0	0.3	1.7	1.5	−0.3	0.0	0.4	1.4	−0.3	−0.1	0.4	0.6
*Difference to gold standard*	6.7	1.8	−3.8	5.0	3.8	8.2	10.1	14.5	2.1	8.0	10.0	13.6
**Post hoc analysis of final OpenStreetMap-derived public open space data, with full exclusion criteria applied as in main study**												
OpenStreetMap (final version)	75.9	58.1	—		54.4	46.0	—	—	77.9	62.7	—	—
Difference to gold standard *	15.5	11.1	—	—	2.7	3.3	—	—	−20.0	−12.7	—	—

*Analysis results for Greater Melbourne presented here used a further refined version of the OpenStreetMap-derived public open space dataset, with analysis restricted for address points located in urban sections of state and employed other exclusion criteria detailed in the methods section.

When considering access to public open space by road network dataset, we found only very marginal differences in estimates for percentage of dwellings with access regardless of typology or open space data source, with these mostly related to fringe areas which were excluded following restriction to the metropolitan urban area. Results using the OpenStreetMap-derived public open space datasets differed by less than 1% for urban areas when using an OpenStreetMap-derived pedestrian network compared to one constructed from the official Vicmap transport dataset. This difference for urban areas due to choice of network dataset was further reduced when revising the OpenStreetMap public open space criteria for representation of public open spaces outside of the the Melbourne setting. This suggests that the use of OpenStreetMap for routing in Australian urban settings like Melbourne is valid, a finding supported by work of other researchers^38^ and supports generalised usage for other urban settings in our study. This preliminary analysis also re-inforced our restriction to address points in Major Urban or Other Urban sections of state.

The magnitude and direction of differences in estimates for access to public open space when using the OpenStreetMap-derived public open space datasets as compared to the VPA ‘gold standard’ varied by class of public open space. The estimates for percentage of urban dwellings with access to any public open space were approximately 13% lower using the final OpenStreetMap-derived dataset than when using the Victorian gold standard dataset. However, access to large public open space was approximately similar; and when considering access to a large public open space, or of any size with a sports facility, estimates were approximately 16% higher when using the final OpenStreetMap-derived dataset. This suggests that while the OpenStreetMap-derived dataset may not have had as comprehensive inclusion of incidental ‘pocket’ or sliver parks and other public open space types, the representation of larger, multipurpose recreational public open spaces was accurate; further, the capacity for querying provision of sporting amenities was far greater using the final OpenStreetMap-derived data.

Estimates for access also varied across iterations of revisions of the method used to derive public open space features. As noted above, revisions of the method were broadly motivated by the application to settings beyond the preliminary Melbourne test setting, where we evaluated identified public open spaces against satellite imagery. As such, our first attempt at re-creating the VPA public open space dataset for Melbourne using OpenStreetMap could be regarded as being over-fit to the Melbourne context, and as we modified the approach to tagging ensure adequate performance in terms of our empirical face validity checks in our other cities the differences from the Victoria-specific data become larger. However, by the final iteration, the important negative difference appeared to be in representation of ‘any’ public open space; while differences to large open space were minimal, capacity to identify specific sport and leisure facilities associated with parks was greatly enhanced using additional information OpenStreetMap sport and leisure-related tags.

We concluded that the broader coverage and more timely representation of open space features in OpenStreetMap meant that in addition to yielding approximately similar results for important scenarios, it was suitable for analysis of access to public open space in urban areas in the absence of other quality, consistent public open space data with national coverage for Australia. Further detail on the implementation of the derived public open space data is provided as supplementary material.

The above sensitivity analysis was focused on a typology of public open space based on a specific set of recommendations made in the Victorian context. Our analysis in the national study was broader than this, however. In the first instance, we analysed the distance to all public open spaces within 3200 metres and to the closest public open space for address points, and allowed for subsequent post hoc querying for specific typologies of relevance to policy or researchers’ interests. The Urban Liveability Index contains a sub-indicator relating to proximal access to a public open space larger than 1.5 hectares that is based upon associations with increased recreational- and overall-walking behaviours in a Melbourne-based cohort^63^, and consequent recommendations^24^. However, we also measured distance to public open space: of any size; with a public toilet within 100 metres; < = 0.4 Ha; >0.4 Ha; >0.5 Ha; >1.5 Ha; >2 Ha; >0.4 to < = 1 Ha; >1 to < = 5 Ha; >5 Ha to < = 20 Ha; >5 Ha; >20 Ha; and having a sport facility. These measures of ‘distance to closest’ are based on typologies having relevance to specific policy settings around Australia^18^, and can be used to derive threshold-based indicators (for example, a Boolean indicator for access within 400 metres). Further, we provide guidance in our supplementary usage notes on GitHub for researchers to define and analyse access to areas of open space using parameters of relevance to their own agenda and research settings. That is, distance, size, attributes and co-locations may be queried as per the examples provided, as required.

### Evaluating access to closest supermarket

Walkable access to a supermarket is an important indicator of a healthy food environment, representing local availability of fresh food, in addition to opportunities for incidental physical activity^16^. In addition to being a measure in its own right, it contributes to the walkability and urban liveability indices in this study, and thus accuracy of measurement was of particular importance. However, evaluation of access to points of interest is contingent on the quality of the data used. Commercial datasets are no guarantee of quality. As a pre-cursor activity to this study, the lead author conducted a review of destination data sources with national scope in 2016, including the Macroplan supermarket data from Pitney Bowes (November 2014), identifying, among other issues that 34 records for Foodworks stores (34/435 = 0.0782 or 7.8% of stores in the dataset) were found to have Y coordinate incorrectly recorded as a linear relationship with X coordinate: Y = -(X/10). At the time these were corrected using locations determined through web-searching. For the 2017 Creating Liveable Cities report which analysed Australia’s capital cities, our research group determined that higher quality contemporary data could be retrieved using web-scraping of major supermarket chains in Australia^24^.

However, when scaling up to Australia’s 21 cities the authors were also aware that independent grocery chains play a major role and were not always captured in the web-scraped data. We hypothesised, then demonstrated, that when determining the distance to closest supermarket the best estimate for individual address locations could be achieved by taking the minimum of their respective estimates using the major chain scraped data (where we assumed that 2017 supermarkets persisted in 2018) and supermarkets identified using OpenStreetMap tags informed through a review of OpenStreetMap TagInfo and Australian tagging guidelines^64,65^ (see Supplementary Table 1). Table 11 summarises the median and interquartile range of estimates for distance to closest supermarket by city and across the data sources: web-scraped; OpenStreetMap-derived; the difference between these estimates; and the row-wise minimum of these two records for each address. The latter is the method we used for evaluating distance to closest supermarket for indicators in the study, to ensure that error for individual address locations due to incompleteness of data was minimised. Overall, the estimates using the two datasets separately were similar, with the median difference being 22 m, slightly in favour of the web-scraped data but with relatively broad interquartile ranges indicating that at least some addresses in each city were better served by accounting for access to a supermarket using the OpenStreetMap data.

**Table 11:** Median and interquartile range of distance (m) to closest supermarket, by city and by data source (ordered by median difference).

City	Median [Interquartile range] of distance (m) to closest supermarket for residential addresses				
	State/Territory	Major chain supermarkets (web scraped)	OpenStreetMap-derived	Difference (Major Chain - OpenStreetMap)	Combined (Closest of either)
Sunshine Coast	Queensland	1,284 [785–1,961]	2,022 [1,187–3,487]	−203 [−1,677, 10]	1,213 [732–1,799]
Wollongong	New South Wales	1,493 [942–2,124]	2,254 [1,265–3,669]	−198 [−1,874, 26]	1,379 [859–2,031]
Townsville	Queensland	1,543 [980–2,367]	2,081 [1,227–4,185]	−154 [−1,233, 6]	1,507 [928–2,338]
Cairns	Queensland	1,595 [1,023–2,434]	2,078 [1,236–3,252]	−123 [−1,084, 74]	1,418 [863–2,117]
Gold Coast – Tweed Heads	Queensland - New South Wales	1,454 [940–2,183]	1,751 [1,135–2,762]	−85 [−558, 14]	1,350 [866–2,047]
Perth	Western Australia	1,207 [806–1,740]	1,587 [988–2,507]	−76 [−773, 8]	1,157 [765–1,682]
Brisbane	Queensland	1,286 [825–1,904]	1,531 [959–2,379]	−53 [−381, 45]	1,184 [753–1,760]
Newcastle - Maitland	New South Wales	1,408 [857–2,161]	1,647 [991–2,703]	−31 [−300, 29]	1,360 [815–2,082]
Albury – Wodonga	New South Wales - Victoria	1,388 [905–1,998]	1,497 [973–2,397]	−22 [−174, 52]	1,292 [848–1,916]
Sydney	New South Wales	1,188 [762–1,746]	1,378 [855–2,057]	−20 [−232, 40]	1,111 [698–1,647]
Melbourne	Victoria	1,151 [742–1,677]	1,269 [809–1,882]	−12 [−171, 55]	1,068 [680–1,567]
Geelong	Victoria	1,237 [790–1,844]	1,273 [809–1,990]	−10 [−76, 34]	1,151 [719–1,770]
Darwin	Northern Territory	1,508 [965–2,264]	1,459 [968–2,068]	−9 [−95, 200]	1,340 [852–1,968]
Hobart	Tasmania	1,597 [895–2,672]	1,613 [874–2,706]	−9 [−104, 110]	1,366 [762–2,243]
Toowoomba	Queensland	1,546 [1,002–2,301]	1,336 [868–2,159]	0 [−76, 122]	1,207 [786–2,005]
Ballarat	Victoria	1,378 [898–1,993]	1,369 [898–1,965]	0 [−47, 52]	1,285 [830–1,847]
Canberra	Australian Capital Territory	1,222 [823–1,701]	1,105 [739–1,575]	8 [−94, 164]	1,001 [668–1,398]
Launceston	Tasmania	1,047 [684–1,616]	855 [551–1,254]	14 [−14, 271]	834 [528–1,234]
Adelaide	South Australia	1,281 [847–1,879]	1,193 [771–1,771]	19 [−96, 328]	1,029 [679–1,458]
Mackay	Queensland	1,595 [1,005–2,807]	1,454 [868–2,231]	23 [−98, 265]	1,359 [808–2,104]
Bendigo	Victoria	2,098 [1,324–3,115]	1,704 [1,092–2,567]	99 [−42, 412]	1,660 [1,053–2,528]
Total		1,241 [800–1,836]	1,398 [876–2,150]	−22 [−240, 56]	1,134 [722–1,683]

The results in Table 11 could also suggest geographic variability of coverage, with access to a supermarket for addresses in regional cities of Australia’s easternmost states (Queensland and New South Wales) performing more strongly using the major chain supermarket dataset overall, while addresses in cities of Australia’s more southern states and territories (South Australia, Victoria, Tasmania and Australian Capital Territory) tending towards better performance using the OpenStreetMap dataset. A plausible explanation could be that the latter cities may contain a greater number of independent supermarkets, or chains other than those selected for in the 2017 major chain web-scraping exercise (Aldi, Coles, Foodworks, IGA and Woolworths)^24^, and were better captured in the OpenStreetMap-derived data. This suggests that by pooling the data the risk of misclassification error for individual addresses when considering access to a supermarket was mitigated, compared to using either of the data sources on their own.

## Usage Notes

We have provided our data and data dictionaries in a plain text CSV format for archival purposes, to maximise accessibility and usability of the data. An Excel file containing the data dictionaries as formatted worksheets is also included. The data dictionaries describe the variables (columns) included in the CSV data, in addition to the data types for interpreting these (e.g., integer, numerical, string or text, etc).

We recommend using an SQL database with appropriate use of indexes to support managing and querying of data. Below we provide example code for the popular free and open-source database management system PostgreSQL (e.g., version 14 or higher) along with the PostGIS extension (e.g., version 3.2.3 or higher) for spatial datatypes and analysis.

A Comprehensive guide to loading and using the data has been provided on GitHub at https://github.com/carlhiggs/Australian-National-Liveability-Study-2018-datasets-supplementary-material. In addition to usage notes, this URL contains machine readable copies of the data dictionaries, metadata, as well as a detailed description of the method used for identifying public open space using OpenStreetMap including SQL code.

The distance array datasets for access to all destinations and areas of open space within 3200 m have records containing comma-separated lists, and so these were respectively saved as zipped pipe-separated values (PSV; 3.4GB zipped) and tab-separated values (TSV; 1.8 GB zipped) files to simplify the process of re-opening and using these files and reduce file size for more manageable storage and download. For each of these datasets unzipped 100-record random sample dataset files were also provided to allow users to preview and trial a subset without needing to access the full datasets. Examples of loading and using these files are also provided in the usage notes hosted on GitHub.

## Supplementary information

Supplementary Table 1

## Data Availability

Code is available on GitHub at https://github.com/healthy-liveable-cities/australian-national-liveability-study. The project was conducted between 2018 and 2020 using Python 2.7 with PostgreSQL 9.6, PostGIS 2.4, the ArcGIS 10.6 arcpy python library and network analyst extension. The code also draws heavily on the psycopg2, sqlalchemy, pandas and osmnx libraries. The code was developed across the duration of the project to meet evolving stakeholder needs for data and indicators. Unfortunately, across this period, software versions also evolved, and when a newer version of ArcGIS was installed in 2020 following expiry and renewal of institutional licences this required the use of Python 3. While this initially provided impetus to re-factor and update the code, project priorities within our research group changed and it became apparent this code would not be used in future projects, and there was not funding or scope to complete final code re-factoring. The exception to this was the ‘highlife’ project branch which contains code developed to create built environment measures targeting 2019 for the separate High Life study; this was the branch with the most recent and complete development efforts, and was therefore set as the default branch for the repository. An incomplete re-factoring for Python 3 is located on the ‘python3_2020 branch’; and the final main working branch of the overall project is the one titled ‘main’. Many lessons were learnt about managing large code projects through the course of the study^8^. The code for this project would ideally be re-factored but no team members had capacity to do so for this completed project. Project experiences meant that the team had broad desire to move towards more open source software solutions, for which the methods developed for this study were adapted and applied in other projects^4,66,67^.
