# Supplementary material for: Policy relevant health related liveability indicator datasets for addresses in Australia’s 21 largest cities
Source: Sci Data. 2023 Feb 25;10:113. doi: 10.1038/s41597-023-02013-5 (PMC9968286; doi:10.1038/s41597-023-02013-5)
Supplement: Supplementary file 1 — Supplementary Table 1 [file 41597_2023_2013_MOESM1_ESM.docx]

Supplementary Table 1. Key-value pair tags used to identify destinations for the Australian National Liveability Study using OpenStreetMap.

| **Destination** | **Key** | **Value** | **2018 Global frequency**  **(OSM taginfo)** | **Combination logic within destination** |
| --- | --- | --- | --- | --- |
| Supermarket | shop | supermarket | 343,085 | OR |
|  | supermarket |  | 95 | OR |
|  | amenity | supermarket | 37 | OR |
|  | building | supermarket | 8,251 | OR |
|  | shop | grocery | 1,137 | OR |
| Bakery | shop | bakery | 154,061 | OR |
|  | shop | pastry | 6,436 | OR |
| Meat / Seafood | shop | butcher | 60,510 | OR |
|  | shop | seafood | 11,651 | OR |
|  | shop | fishmonger | 1,029 | OR |
| Fruit and Veg | shop | greengrocer | 33,791 | OR |
|  | shop | fruit | 50 | OR |
|  | shop | fruits | 22 | OR |
|  | shop | vegetables | 10 | OR |
| Deli | shop | deli | 12,735 | OR |
|  | shop | cheese | 2,415 | OR |
| Convenience | shop | convenience | 457,453 | OR |
|  | amenity | fuel | 398,945 | OR |
|  | shop | kiosk | 72,391 | OR |
|  | shop | newsagent | 19,245 | OR |
|  | shop | newsagency | 10 | OR |
|  | amenity | newsagency | 3 | OR |
| Other food | shop | food | 1,956 | OR |
| Health food | shop | health_food | 816 | OR |
| Market | amenity | marketplace | 52267 | OR |
|  | amenity | market | 206 | OR |
|  | amenity | market_place | 41 | OR |
|  | amenity | public_market | 22 | OR |
|  | shop | marketplace | 248 | OR |
|  | shop | market | 246 | OR |
| Community centre | community_centre |  | 3,392 | OR |
|  | amenity | community_centre | 86,020 | OR |
|  | amenity | social_centre | 2,082 | OR |
| Place of Worship | amenity | place_of_worship | 1,036,820 | OR |
|  | place_of_worship |  | 2,357 | OR |
|  | building | church | 243,577 | OR |
|  | building | chapel | 49,037 | OR |
|  | building | mosque | 32,690 | OR |
|  | building | temple | 7,382 | OR |
|  | building | shrine | 1,872 | OR |
| Museum | tourism | museum | 73,881 | OR |
| Theatre | amenity | theatre | 31,943 | OR |
| Cinema | amenity | cinema | 24,284 | OR |
| Art gallery | tourism | gallery | 5,503 | OR |
| Art centre | amenity | arts_centre | 17,542 | OR |
| Artwork | tourism | artwork | 97,466 | OR |
| Fountain | amenity | fountain | 100,217 | OR |
| Viewpoint | tourism | viewpoint | 139,617 | OR |
| Picnic site | tourism | picnic_site | 114,350 | OR |
| Pharmacy | amenity | pharmacy | 253,978 | OR |
|  | amenity | chemist | 15 | OR |
|  | shop | pharmacy | 53 | OR |
|  | shop | chemist | 2,495 | OR |
| restaurant | amenity | restaurant | 924,972 | OR |
| cafe | amenity | cafe | 356,890 | OR |
| eatery | cuisine |  | 394,005 | OR |
| food_court | amenity | food_court | 6,309 | OR |
| Fast food | amenity | fast_food | 326,229 | OR |
| Pub | amenity | pub | 151,404 | OR |
| Bar | amenity | bar | 156,117 | OR |
| Nightclub | amenity | nightclub | 18,994 | OR |
| Gambling | amenity | casino | 5,327 | OR |
|  | amenity | gambling | 2,300 | OR |
|  | shop | bookmaker | 7,350 | OR |
|  | gambling |  | 1,678 | OR |
| Alcohol outlet | shop | alcohol | 47,756 | OR |
| Tobacconist | shop | tobacco | 12,150 | OR |
| Tobacconist | shop | tobacconist | 20 | OR |
| Post office | amenity | post_office | 156,584 | OR |
| Public swimming pool | leisure | sports_centre | 184 490 | AND |
|  | leisure | swimming_pool | 978,090 | OR |
|  | amenity | swimming_pool | 18,885 | OR |
|  | landuse | swimming_pool | 26 | OR |
|  | sport | swimming_pool | 24 | OR |
|  | sport | swimming | 84,522 | OR |
|  | water | swimming_pool | 83 | OR |
|  | swimming_pool | yes | 726 | OR |
|  | access | private | *Not audited* | NOT |
